# Age-related morphological regression of myelinated fibers and capillary architecture of distal peripheral nerves in rats

**DOI:** 10.1186/s12868-016-0277-4

**Published:** 2016-06-24

**Authors:** Masahiro Sakita, Shinichiro Murakami, Hidemi Fujino

**Affiliations:** Department of Physical Therapy, Faculty of Health Sciences, Kyoto Tachibana University, 34 Oyakeyamada, Yamashina Ward, Kyoto City, Kyoto 607-8175 Japan; Department of Physical Therapy, Faculty of Health Care Sciences, Himeji-Dokkyo University, 7-2-1 Kamiono, Himeji City, Hyogo 670-0896 Japan; Department of Rehabilitation Science, Kobe University Graduate School of Health Sciences, 7-10-2 Tomogaoka, Suma Ward, Kobe City, Hyogo 654-0412 Japan

**Keywords:** Myelinated fiber, Capillary, Tibial nerve, Three-dimensional image

## Abstract

**Background:**

Regression of myelinated peripheral nerve fibers in the lower extremities contributes to sarcopenia and balance dysfunction in normal aging. This subclinical regression of myelinated fibers (MFs) is heavily influenced by alterations in microvasculature, though the mechanism underlying these age-related degenerative phenomena remains unclear. The aim of the present study was to examine age-related regressions in myelinated distal peripheral nerve fibers as well as capillary architecture in rats using both morphological and histochemical methods.

**Results:**

MFs were categorized into tertiles of ‘large’, ‘medium’, and ‘small’ sizes based on the distribution of MF diameters. A two-way ANOVA was used to assess effects of fiber size (large/medium/small) and group (young/elderly) on myelin thickness, axon diameter, myelin perimeter, axon perimeter, and G-ratio (axon diameter/fiber diameter). Significant main effects were observed for both MF size and group with respect to all dimensions except for G-ratio. Values for fiber diameter (P < 0.01), myelin thickness (P < 0.01), axon diameter (P < 0.01), myelin perimeter (P < 0.01), and axon perimeter (P < 0.01) were significantly lower than those in the young group. Additionally, mean capillary diameter and number of microvascular branch points were significantly lower in the elderly group than in the young group.

**Conclusions:**

The present study demonstrated that spontaneous age-related regression predominantly occurs for all fiber sizes in the distal peripheral nerves and the capillary architecture. The results of the present study further suggest that both the distal MFs and capillaries in the peripheral nerve may simultaneously regress with aging.

## Background

Sarcopenia refers to the degenerative loss of skeletal muscle mass, muscle fibers, and strength associated with normal aging and is the main risk factor for falls in the elderly [1–4]. Research has further revealed that myelinated fibers (MFs) of the peripheral nerves also spontaneously degenerate with age [5–7]. Histological studies have demonstrated that aging is associated with a decrease in the number of MFs and increased atrophy of MFs in the dorsal root ganglia of the lumbar spinal cord [8] and sciatic nerve [7, 9] in mice. In particular, a recent animal study found that atrophy of the peripheral nerve occurs prior to age-related sarcopenia [10]. Thus, it may be dysfunction of the peripheral nerve rather than sarcopenia itself that contributes to falls experienced by elderly people.

Capillary regression precedes age-related neurodegeneration in the cerebral microvasculature [11, 12]. Structural preservation and capillary angiogenesis are promoted by vascular endothelial growth factor (VEGF), which is secreted to induce the sprouting and subsequent differentiation of capillaries [13]. VEGF activity promotes the formation and maintenance of the endothelial cells that form the inner lining of capillaries and has also been associated with the pathogenesis of chronic nerve compression. Namely, in situ hybridization studies have revealed that the production of VEGF mRNA by Schwann cells underlies increases in vascularity observed in response to chronic nerve compression, though it is unclear whether similar increases in Schwann cell VEGF occur spontaneously in the normal aging process [14]. Furthermore, Schwann cell-derived VEGF in the peripheral nerves has been observed to promote arteriogenesis and differentiation of primitive capillaries in vivo [15].

Both in vivo and in vitro studies have revealed that the production of brain-derived neurotrophic factor (BDNF) is induced by endothelial cells [16, 17]. BDNF is a ligand that promotes the survival and formation of myelin sheaths. Nerve growth factor (NGF), which promotes extension of neurites and axonal outgrowth, is secreted by non-neural Schwann cells and fibroblasts in highly-pure, serum-free conditions [18]. Considering the association of their functions, an interdependent relationship between MFs and capillaries in the peripheral nerves is likely.

By the same rationale, it can be proposed that there is an age-related reduction in cross-talk (Schwann cell production of VEGF, endothelial cell production of NGF, and BDNF) between MFs in the peripheral nerves and capillaries. However, little is known about whether MFs and capillaries of distal peripheral nerves undergo simultaneous age-related morphological regressions. Thus, the aim of the present study was to examine potential mechanisms underlying these age-related regressions using both morphological and histochemical methods.

## Methods

### Animals

Six male Wistar rats (Japan SLC, Inc.) were used in the experiment. The rats were acquired at 10 weeks of age and maintained until 20 weeks of age (young group), with a subset of 3 randomly selected rats maintained until 90 weeks of age (elderly group). Each rat was housed in a cage (two rats per cage) with a 12-h light and dark cycle at a temperature of 22 ± 2 °C with 40–60 % humidity. Food and water were provided ad libitum.

### Nerve sample preparation

Rat body mass in the young and elderly groups was measured at 20 and 90 weeks of age, respectively. The rats were then anesthetized with pentobarbital sodium (50 mg/kg; i.p.). A catheter containing contrast medium was inserted following opening of the abdominal cavity and ligation of the aorta to maintain perfusion of the bilateral hind limbs. The blood vessels in the bilateral hind limbs were perfused with a 0.9 % physiological saline solution containing 10,000 IU/L of heparin at 37 °C for three minutes to wash out the intravascular blood. Then, contrast medium solution consisting of 10 % glucose, 1 % fluorescent material (PUSR80; Mitsubishi Pencil, Tokyo, Japan), 8 % gelatin (Nakalai Tesque, Kyoto, Japan), and distilled water was injected into the aorta to fill the microvasculature in both hind limbs under a perfusion pressure of 120 mmHg [19]. Immediately after perfusion with contrast medium, the whole body of each rat was immersed in cold saline for 20 min. A series of nerves from the sciatic L4–L5 anastomosis leading to the distal portion of the tibial nerve that branches into the medial and lateral planter nerves were removed in the bilateral hind limbs. The removed nerves were then frozen in isopentane precooled in liquid nitrogen. The nerves were stored at −80 °C until the histochemical staining procedure.

### Histochemical processes

The removed frozen nerves were cut at a distance of 3 mm from the distal portion, and the cut sample block (6 blocks; young group, 6 blocks; elderly group) was infused with Tissue Tek OCT compound in an embedding container. The embedded block was then frozen in liquid nitrogen. Serial transverse semi-thin sections (5 μm in thickness) were cut using a cryostat microtome (CM3050; Leica Microsystems, Mannheim, Germany) at −20 °C. The sections were then allowed to thaw at room temperature and air-dry for 15 min. Three to four sliced sections per cut nerve were stained with 1 % toluidine blue solution (1 g toluidine blue and 100 ml distilled water) for visualization of myelin sheaths in the peripheral nerves. The semi-thin sections were first deparaffinized with 100 % xylene, which was repeated three times every 4 min. In order to remove the xylene, sections were immersed once in 80 % alcohol and then in 100 % alcohol three times every 3 min. The sections were then washed three times with distilled water and subsequently stained with 1 % toluidine blue solution for 10 min. Stained sections were quickly dehydrated by immersion in 100 % alcohol three times followed by immersion in 100 % xylene three times every 4 min. The dehydrated sections were then enclosed with mounting medium and covered with a thin coverslip.

**Fig. 1:**
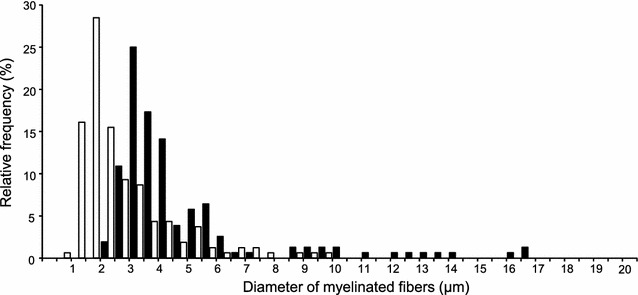
Histogram depicting the distribution of the diameter of myelinated fibers in the distal tibial nerve of young rats (*black bars*) and elderly rats (*white bars*). *Note* that a number of small myelinated fibers for the elderly group are larger than those of the young group, which indicates that the histogram for the elderly group shifted to the left in comparison with that of the young group (P < 0.01)

The semi-thin sections were observed using a light microscope (BX-53; Olympus, Tokyo, Japan) with a ×100 objective lens. Three to four section images per cut nerve were synchronously captured by a CMOS (complementary metal-oxide semiconductor); camera (Moticam 3; Shimadzu, Kyoto, Japan) and a personal computer, which expanded the image to a magnification of ×288. At least ten MFs in each captured image were randomly selected for observation by a blind observer. For calculation of the G-ratio (axon diameter/fiber diameter) [20], fiber diameter, axon diameter, and myelin thickness [(fiber diameter − axon diameter)/2] [21] were measured (at least 9 images per group) using NIH image software (ImageJ version 1.48). Fiber and axon diameters were calculated as the mean values of maximum and minimum diameter lines drawn via a virtual center of mass in transverse MF sections. A centroid function was used to determine the virtual center of mass, which was defined as the average of the x and y coordinates of all pixels within the traced outline of the myelin sheath. In addition, outer boundaries of the myelin sheath and axons were traced, and their perimeters were calculated [22]. Then, fiber diameter data for both the young and elderly groups were respectively split into three quantiles (large, medium, and small MFs) using the histograms presented in Fig. 1. The mean values of myelin thickness, axon diameter, myelin perimeter, axon perimeter, and G-ratio for each group were then calculated based on the three fiber diameter quantiles.

### Analysis of capillary architecture

The three-dimensional (3-D) structure of capillaries in the tibial nerve was observed using a confocal laser microscope (CLM) (TCS-SP; Leica Instruments, Mannheim, Germany) an argon laser (488 nm) in fluorescent mode [23, 24]. Frozen nerve samples that had been cut 3 mm from the distal end were fixed with 4 % paraformaldehyde (pH 7.4). The sample block (6 blocks; young group, 6 blocks; elderly group) were sliced into 80 μm sagittal sections using a cryostat and allowed to thaw at room temperature for 10 min. CLM images were obtained using a ×20 objective lens. Each section was scanned to 50 μm depths at 1 μm/slice, and the CLM images were stacked as 3-D images with a depth of 50 μm. In three to four stacked 3-D images per section, ten capillaries less than 10 μm diameter [25, 26] were randomly chosen and microvascular branch points [25, 26] were counted on images measuring 800 × 800 μm^2^ in area using the ImageJ version 1.48 software program.

### Statistical analysis

A Mann–Whitney U-test was performed to compare body mass and MF diameter between the young and elderly groups. A two-way (3 fiber sizes × 2 groups) analysis of variance (ANOVA) was used to compare the effect of fiber size (small/medium/large) and group (young/elderly) on fiber diameter, myelin thickness, axon diameter, myelin perimeter, axon perimeter, and G-ratio. For significant main effects and/or interactions, the Tukey–Kramer post hoc test was used. P-values of less than 0.05 (P < 0.05) were considered statistically significant.

## Results

### Distribution of MF diameter in distal tibial nerves

No significant difference in body mass was observed between the young (median and quartile range; 434.30 and 29.50 g) and elderly groups (median and quartile; 465.60 and 60.40 g) Fiber diameters in the young and elderly groups were respectively calculated using the mean value of maximum and minimum diameter lines that pass through a virtual center of mass in transverse MF sections. The fiber diameter data for both groups were then categorized into tertiles (large, medium, and small MFs) based on the histograms presented in Fig. 1. MF fiber distribution in the elderly group (median and quartile range: 2.12 and 1.74 μm) decreased in comparison to that of the young group (median and quartile range: 3.81 and 1.89 μm) (P < 0.01).

### Characteristics of MF fiber sizes in the young and elderly groups

**Fig. 2:**
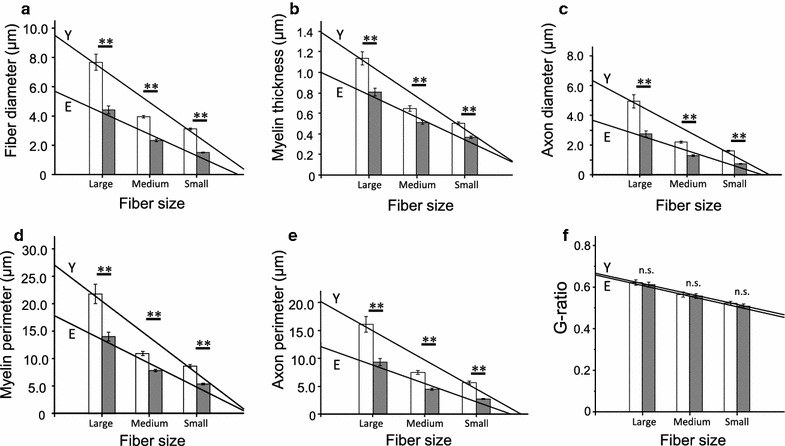
Comparison between the young and elderly groups regarding characteristics of myelinated fiber types. The elderly group had a smaller fiber diameter (**a**), myelin thickness (**b**), axon diameter (**c**), myelin perimeter (**d**), and axon perimeter (**e**) for all MF sizes (large, medium, and small) in when compared with the young group (**f**). No significant differences between the young and elderly groups were observed for any fiber size with respect to G-ratio. *Open* and *filled* columns in each graph indicate mean values of large, medium, and small myelinated fiber sizes. *Y* and *E* in each graph indicate fitted regression lines for the young and elderly groups, respectively (See Table 1 to confirm the regression coefficients and intercepts related to regression lines of the young and elderly groups with respect to each factor). Values are presented as mean ± SEM. ** P < 0.01, young versus elderly. *MF* myelinated fiber

**Fig. 3:**
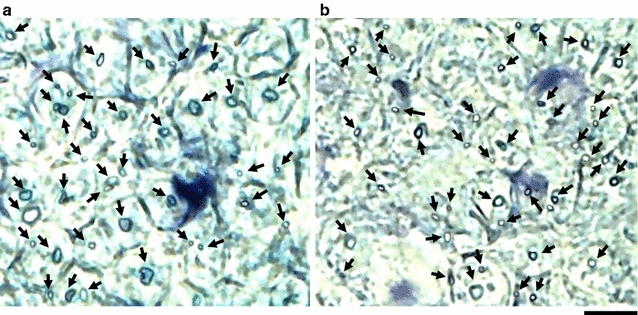
Cross-sectional images of the tibial nerve stained with 1 % toluidine blue. The *black arrows* indicate myelinated fibers. Each densely stained outer ring indicates a myelin sheath. The myelin sheaths and axons of the myelinated fibers in a young rat appear to be thicker and larger than those of an elderly rat (**a** a young rat, **b** an elderly rat). *Scale bar* 10 μm

There were significant major effects of fiber size on fiber diameter [*F*(2, 313) = 122.76, P < 0.0001], myelin thickness [*F*(2, 313) = 137.57, P < 0.0001], axon diameter [*F*(2, 313) = 97.96, P < 0.0001], myelin perimeter [*F*(2, 313) = 101.66, P < 0.0001], axon perimeter [*F*(2, 313) = 94.72, P < 0.0001], and G-ratio [*F*(2, 313) = 40.23, P < 0.0001]. There were also significant effects of age group on fiber diameter [*F*(1, 313) = 112.47, P < 0.0001], myelin thickness [*F*(1, 313) = 53.74, P < 0.0001], axon diameter [*F*(1, 313) = 63.87, P < 0.0001], myelin perimeter [*F*(1, 313) = 51.48, P < 0.0001], and axon perimeter [*F*(1, 313) = 62.87, P < 0.0001], but not for G-ratio (P = 0.27). The interaction between fiber size and age group also had an effect on fiber diameter [*F*(2, 313) = 7.02, P = 0.0011], myelin thickness [*F*(2, 313) = 5.51, P = 0.0045], axon diameter [*F*(2, 313) = 6.93, P = 0.0012], myelin perimeter [*F*(2, 313) = 5.38, P = 0.0051], and axon perimeter [*F*(2, 313) = 5.70, P = 0.0037], but not on G-ratio (P = 0.96). All major effects are presented in Table 1, along with interactions, regression coefficients, and regression line intercepts for both the young and elderly groups. Post hoc analysis indicated that there were significant differences between the young and elderly groups with respect to fiber diameter (P < 0.01 for large, medium, and small) (Fig. 2a), myelin thickness (P < 0.01 for large, medium, and small) (Fig. 2b), axon diameter (P < 0.01 for large, medium, and small) (Fig. 2c), myelin perimeter (P < 0.01 for large, medium, and small) (Fig. 2d), and axon perimeter (P < 0.01 for large, medium, and small) (Fig. 2e), but not with respect to G-ratio (Fig. 2f). Specifically, cross-sectional images of the tibial nerve stained with toluidine blue solution revealed that the myelin sheaths and axons of MFs in elderly rats (Fig. 3a) were thinner than those observed in young rats (Fig. 3b).

**Table 1:** Characteristics of the three myelinated fiber sizes in the young and elderly groups

Fiber size	Large myelinated fiber		Medium myelinated fiber		Small myelinated fiber		Regression coefficient		Intercept		R^2^
Group	Young (20 weeks old)	Elderly (90 weeks old)	Medium myelinated fiber	Elderly (90 weeks old)	Young (20 weeks old)	Elderly (90 weeks old)	Young (20 weeks old)	Elderly (90 weeks old)	Young (20 weeks old)	Elderly (90 weeks old)	
Fiber diameter (μm)^a,b,c^	7.67 ± 0.54	4.42 ± 0.27**	3.96 ± 0.09	2.33 ± 0.09**	3.12 ± 0.06	1.50 ± 0.04**	−2.27	−1.46	9.46	5.67	0.54
Myelin thickness (μm)^a,b,c^	1.13 ± 0.06	0.81 ± 0.04**	0.65 ± 0.03	0.51 ± 0.01**	0.50 ± 0.01	0.37 ± 0.01**	−0.32	−0.22	1.40	1.00	0.52
Axon diameter (μm)^a,b,c^	5.02 ± 0.45	2.81 ± 0.21**	2.23 ± 0.07	1.31 ± 0.06**	1.63 ± 0.05	0.76 ± 0.03**	−1.69	−1.02	6.34	3.67	0.46
Myelin perimeter (μm)^a,b,c^	21.85 ± 1.76	14.08 ± 0.80**	10.99 ± 0.38	7.89 ± 0.22**	7.69 ± 0.26	5.41 ± 0.13**	−6.57	−4.34	26.99	17.80	0.47
Axon perimeter (μm)^a,b,c^	16.06 ± 1.40	9.29 ± 0.65**	7.43 ± 0.35	4.46 ± 0.19**	5.63 ± 0.29	2.70 ± 0.08**	−5.21	−3.3	20.12	12.07	0.46
G-ratio^a^	0.62 ± 0.01	0.61 ± 0.01	0.56 ± 0.01	0.56 ± 0.01	0.52 ± 0.01	0.51 ± 0.01	−0.05	−0.05	0.67	0.66	0.21

Fiber diameter, myelin thickness, axon diameter, myelin perimeter, axon perimeter and G-ratio of the young and elderly groups for the three myelinated fiber sizes (large, medium, and small myelinated fibers). Regression coefficients and intercepts indicate those of regression lines on total fiber diameter, myelin thickness, axon diameter, myelin perimeter, axon perimeter, and g-ratio for the young and elderly groups. R^2^ indicates determination coefficient of a two-way ANOVA

** Significant difference between the young and elderly groups with Tukey–Kramer post hoc test (P < 0.01). Values of fiber diameter, myelin thickness, axon diameter, myelin perimeter, axon perimeter and g-ratio for the young and elderly groups according to fiber size (large, medium, and small myelinated fibers) are presented as mean ± SEM

^a^Main effect on the size myelinated fiber with a two-way ANOVA (P < 0.0001)

^b^Main effect on the group with a two-way ANOVA (P < 0.0001)

^c^Interaction of the size myelinated fiber and group with a two-way ANOVA (P < 0.01)

### Distribution of capillary diameter in the distal tibial nerve

**Fig. 4:**
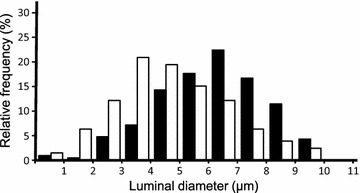
Histogram indicating the distribution of capillary diameter in the distal tibial nerve of young (*black columns*) and elderly (*white columns*) rats. *Note* that a number of small capillaries for the elderly group are larger than that of the young group, which indicates that the histogram for the elderly group shifted to the left with respect to that of the young group

Regarding the luminal diameter distributions of the capillaries for the young and elderly groups, the histogram for the elderly group (median: 4.63 μm; skewness: 0.28) was shifted to the left when compared to that of the young group (median: 6.24 μm; skewness: −0.28), indicating a greater number of small capillaries in the elderly group than in the young group (Fig. 4).

### Three-dimensional analysis of microvasculature and capillary diameter

**Fig. 5:**
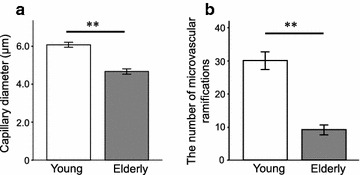
Comparison between the young and elderly groups in capillary diameter and microvascular branch points of the intra-tibial nerve. **a** Mean capillary diameter of the elderly was significantly smaller in comparison to that of the young group (P < 0.01). **b** The mean number of microvascular branch points for the elderly group was significantly decreased in comparison to that observed for the young group (P < 0.01). Values are presented as mean ± SEM. ** P < 0.01, young versus elderly

**Fig. 6:**
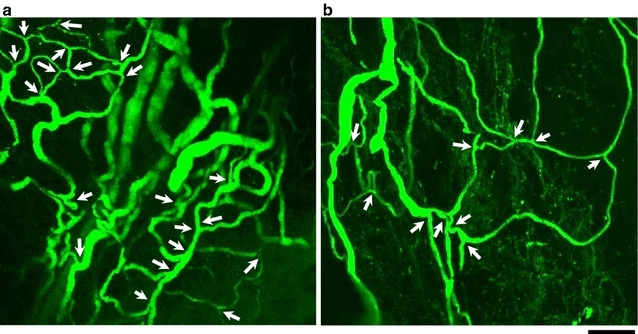
The three-dimensional architecture images of capillaries in the tibial nerve captured by confocal laser scanning microscopy. Each stacked image represents a sagittal view. *Upper* and *lower parts* of the images correspond to the proximal and distal parts of the distal tibial nerve, respectively. The *white arrows* indicate the microvascular ramifications. The capillaries of the intra-tibial nerve in a young rat seemed to be more compact than those of an elderly rat (**a** a young rat, **b** an elderly rat). *Scale bar* 100 μm

Both the capillary diameter (young group: 6.08 ± 0.13 μm; elderly group 4.67 ± 0.14 μm; P < 0.01) (Fig. 5a) and number of microvascular branch points (young group: 30.07 ± 2.62; elderly group 9.17 ± 1.83; P < 0.01) (Fig. 5b) of the elderly group were significantly smaller than those of the young group. Visual inspection of the generated 3-D images (Fig. 6) revealed that the capillary architecture of elderly rats had noticeably regressed and contained fewer branches than that of young rats.

## Discussion

The aim of the present study was to examine spontaneous age-related regressions in both MFs and capillary architecture of rat distal peripheral nerves. When compared to rats in the young group, rats in the elderly group had a smaller fiber diameter, myelin thickness, axon diameter, myelin perimeter, and axon perimeter in large, medium, and small MFs. Further, the capillary diameter was smaller and the number of microvascular branch points lower in the elderly group than in the young group. To the best of our knowledge, the present study provides the first evidence for age-related regression in all fiber sizes and capillaries in the distal peripheral nerves.

Longitudinal studies have shown that, between 24 to 108 weeks of age, MF shape in the sciatic nerves of mice indicates maturation at 48 weeks of age. Namely, greater fiber size generally represents the end of the adult maturation period. After the animals have reached 48 weeks of age, functional changed in MFs gradually impair nerve conduction velocity, following which morphological changes in MFs occur [5, 8]. The loss and atrophy of small (<9 μm in myelin perimeter), medium (9–15 μm in myelin perimeter) and large (15–23 μm in myelin perimeter) MF fibers appear to reflect a parallel process of axonal atrophy [8]. The regression of older myelinated distal tibial nerve fibers may be related to the failure of the axonal transport system and reductions in conduction velocity to neurofilaments (NFs) [8, 27–29] responsible for the transport of various proteins involved in maintaining the cytoskeletal framework [29]. The axons at distal MF sites in particular may be more susceptible to age-related regression due to progressive abnormalities in neurofilaments.

Myelin sheath thickness for all three MF sizes in the elderly group was noticeably reduced in comparison with that observed in the young group. Previous research has revealed noticeable decreases in mRNA and protein levels for myelin sheath components in the sciatic nerves of older rats [30]. The reduction in myelin sheath thickness in the distal tibial nerve might therefore be influenced by the incomplete expression of these proteins.

Mean myelin perimeter values of both the young and elderly groups for each fiber size (small/medium/large) divided by fiber diameters were similar to those obtained for previous MF classifications (>23 μm for large MFs, 15–23 μm for medium MFs, and <9 μm for small MFs; see Table 1 and Fig. 3) [7]. Such a result indicates that the myelin sheaths and axons of distal MFs of all three fiber sizes underwent atrophy in the elderly group. This finding is further supported by the observation that G-ratios for all three fiber sizes were not significantly different between the young and elderly groups. Ceballos et al. investigated age-related morphological changes at the proximal site of the tibial nerve in mice and found a significant decrease in the number of medium and small size MFs with age [7]. The authors predicted that the reduction in MF number at the proximal site of the tibial nerve occurs at a later age and is preceded by a loss of MFs at more distal segments. The age-related atrophy and apoptosis seen in distal MFs might be explained by the fact that the distance required for axonal transport is greater for the distal site than the proximal site.

Capillary architecture has been previously assessed based on the number of capillaries per unit area of cross-sectional tissue (capillary density) and the number of capillaries per individual fiber (capillary-to-fiber ratio), using two-dimensional images [31–33]. However, some recent studies have established a method of analysis that uses 3-D images of capillary architecture in the peripheral nerve and muscle that are generated by CLM, and have measured actual capillary diameter and anastomosis [23, 25, 34]. With reference to these findings, we observed that capillary diameter and the number of microvascular branch points in the elderly group were lower than those observed in the young group. Previous investigations have revealed that the initiation of cell death is related to increments in reactive oxygen species (ROS) [35–37] and age-related declines in hypoxia inducible factor-1 (HIF-1) reactivity. HIF-1 is a transcriptional factor responsible for the promotion of VEGF expression and is therefore implicated in the subsequent promotion of angiogenesis [38]. The results of the present study further support the idea that such regressions may be induced by gradual declines in ROS and HIF-1.

Of particular interest is the finding that MFs and capillaries in the distal peripheral nerves spontaneously regressed with age. Schwann cells and axons affected by metabolic dysfunction suppress the expression of VEGF [15], which leads to capillary regression. Moreover, research has revealed that BDNFs that promote the survival and formation of myelin sheaths of Schwann cells are secreted by endothelial cells [16, 17], and that NGFs that promote the extension of neurites and axonal outgrowth and formation are secreted by Schwann cells [18, 39]. Age-related failure of cross-talk among these proteins may thus cause synchronous regressions in both distal MFs and capillaries.

## Conclusion

In conclusion, the present study provides evidence regarding the spontaneous age-related degeneration of capillaries and atrophy of MFs in the distal peripheral nerves, particularly with respect to the axon and myelin sheath. Results suggest that metabolic dysfunction and failure of cross-talk between the distal MFs and capillaries may be responsible for the observed synchronous regressions. Therefore, future studies should investigate VEGF, BDNF, and NGF expressions during aging using immunohistochemical and biochemical techniques.

## Abbreviations

BDNF: brain-derived neurotrophic factor
CMOS: complementary metal-oxide semiconductor
CLM: confocal laser microscope
HIF-1: hypoxia inducible factor-1
MF: myelinated fiber
NF: neurofilament
NGF: nerve growth factor
ROS: reactive oxygen species
VEGF: vascular endothelial growth factor
